# Clinical outcomes of primary surgical treatment for acquired vulvar lymphangioma circumscriptum

**DOI:** 10.1007/s00404-015-3801-3

**Published:** 2015-07-09

**Authors:** Gun Yoon, Hyun-Soo Kim, Yoo-Young Lee, Tae-Joong Kim, Chel-Hun Choi, Byoung-Gie Kim, Duk-Soo Bae, Ji Hye Hwang, Jeong-Won Lee

**Affiliations:** Department of Obstetrics and Gynecology, Pusan National University Yangsan Hospital, Pusan National University School of Medicine, Yangsan, Republic of Korea; Department of Pathology, Severance Hospital, Yonsei University College of Medicine, Seoul, Republic of Korea; Department of Obstetrics and Gynecology, Samsung Medical Center, Sungkyunkwan University School of Medicine, 81, Irwon-ro, Gangnam-gu, Seoul, 135-710 Republic of Korea; Department of Physical and Rehabilitation Medicine, Samsung Medical Center, Sungkyunkwan University School of Medicine, 81, Irwon-ro, Gangnam-gu, Seoul, 135-710 Republic of Korea

**Keywords:** Vulva, Lymphangioma circumscriptum, Surgery, Cervical cancer, Radical hysterectomy, Radiation therapy

## Abstract

**Objective:**

To assess the clinical outcomes of surgical treatment for acquired vulvar lymphangioma circumscriptum in patients who received radical surgery and/or adjuvant radiation therapy for cervical cancer.

**Methods:**

A retrospective chart review of eight patients was performed to assess the demographic information, chief complaints, treatment modality for cervical cancer, location, and primary treatment modality for vulvar LC, postoperative changes in symptoms, and/or signs, the development of local recurrence and the outcome of patients.

**Results:**

All eight patients were previously diagnosed with cervical cancer FIGO clinical stage IA to IIA and received surgery, radiation therapy, or concurrent chemoradiation therapy. Microscopic examination revealed multiple, dilated, D2-40-positive dermal vascular channels containing eosinophilic proteinaceous material, consistent with LC. Most chief complaints showed considerable improvements on assessment at the outpatient clinic after the primary surgery. No patient showed aggravation of symptoms. Two patients developed local recurrences. One patient developed recurrence on the opposite side 13 months after local excision. We performed a second wide local excision. Another patient developed recurrence 47 months after the primary surgery. Since the lesion was very small and localized, we decided to manage it conservatively, but monitor it very closely. The remaining six patients remained free of recurrence.

**Conclusion:**

It is not easy for gynecologists to have an initial clinical diagnosis of LC, because there are a number of diseases that exhibit similar clinical manifestation to that of vulvar LC. Even if it is diagnosed correctly, local recurrence often occurs. Relevant symptoms associated with LC are not only distressing, but also affect patients’ quality of life. Based on our data, we propose that surgical treatment could provide a more long-lasting answer compared to other treatment modalities, since it is beneficial in terms of clinical outcomes. In the future, a long-term follow-up investigation is required to assess the prognosis and to compare the efficacy and side effects of each modality.

## Introduction

Lymphangioma circumscriptum (LC) is a benign disease of nonspecific origin. It occurs in the lymphatic vascular system in the deep dermal and subcutaneous layer. It was initially reported by Fox and Fox in 1878 [1], and the term LC was first used by Malcolm and Morris in 1889 [2]. The pathogenesis of LC is unclear. Usually, it often occurs in the proximal part of limbs, which is rich in lymphatic vasculature such as shoulders, axilla, groin, buttock, and so on. Even though primary vulvar LC can occur congenitally (or primarily) due to the developmental defect of the vulvar lymphatic system, it is a very rare condition [3]. It can also be acquired (or secondarily) in case of cervical cancer patients who received radical hysterectomy, pelvic lymphadenectomy, or pelvic radiation that can damage the lymphatics [4]. The linkage between the presentation of LC as superficial verrucous vesicles and deep lymphatic vasculature was suggested [5].

The symptoms of LC are vulvar swelling, pain, pruritus, eczematous change, and infection. It is cosmetically problematic and deeply distressing. Therefore, it often affects patients’ quality of life. When it gets severe, LC can also affect social and sexual life and produce psychological problems as well [6]. Therefore, the aim of treatment includes palliation for symptomatic relief.

Since vulvar LC typically presents as multiple, grossly verrucous vesicles of various sizes, it may be impossible to distinguish vulvar LC clinically from herpes zoster, condyloma acuminatum, genital warts, molluscum contagiosum, or lupus verrucosus. In this sense, biopsy is essential to confirm the diagnosis of LC.

Although diverse treatment modalities of LC have been attempted, recurrence is a constant problem for gynecologists. The purpose of this study was to demonstrate and assess the clinical outcome of surgical treatment for acquired vulvar LC.

## Methods

We found 12 patients who were diagnosed with vulvar LC at the Department of Obstetrics and Gynecology, Samsung Medical Center (Seoul, Republic of Korea) from January 2005 to December 2014. In all 12 patients, the diagnosis of vulvar LC was confirmed histopathologically. Eight of these patients received surgical excision. Among four patients who received nonsurgical treatments, one did not want to receive surgical treatment. She received CO_2_ laser treatment four times at the Department of Dermatology, Samsung Medical Center (Seoul, Republic of Korea) from July 2012 to December 2012. The remaining three patients had focal and unilateral lesions. Therefore, we only performed vulvar excisional biopsy for them. They are in the process of uneventful clinical follow-up. These patients who did not receive primary surgical treatment were excluded from this study. To assess the clinical outcome after the primary surgery of vulvar LC, we reviewed the medical chart of the outpatient clinic and confirmed the collected information using a person-to-person phone survey for each patient. A retrospective chart review was performed to assess the demographic information, chief complaints, treatment modality for cervical cancer, location of vulvar LC, primary treatment modality for vulvar LC, postoperative changes in symptoms and/or signs, the development of local recurrence, and the outcome of patients. Based on the numeric rating scale (NRS), we designated the symptoms and/or signs as “improved” when there were more than four changes in scale before and after the surgery. We also assessed the remaining clinical parameters using the NRS method. When it was outside of this four scale boundary, we designated it as “stationary”.

## Results

The clinical profiles and surgical outcomes are summarized in Table 1. The clinical stages of the eight patients were FIGO stage IIA in four, IB in three and IA in one patient. All patients received radical abdominal hysterectomy with bilateral pelvic lymph node dissection. Four of them also received radiation therapy and two received concurrent chemoradiation therapy. Therefore, all of them developed acquired (or secondary) type of vulvar LC. The median age of patients and age at presentation were 61.5 and 54 years, respectively. The mean interval between the surgery for cervical cancer and the development of vulvar LC was 17 years (range 7–31 years). To determine the clinical outcome of surgical treatment, we chose the symptoms and/or signs that the patients felt most uncomfortable or distressing. Chief complaints included pain, edema, pruritus, discharge, and secondary infection, according to the frequency. Except for patient 3, who received local excision for unilateral, localized vulvar LC, all had bilateral lesions. Wide local excision was performed in seven patients.

**Table 1:** Clinical profiles and surgical outcomes after primary treatment in eight cases of vulvar lymphangioma circumscriptum

Patient no.	Age/age at presentation	Chief complaints	Previous diagnosis	Treatment for Cx Ca	Type	Location	Ttreatment for LC	Changes in symptoms and signs after treatment for LC					Recurrence/treatment
								Pain	Pruritus	Edema	Discharge	Infection	
1	62/51	Pain, pruritus, edema, discharge	Cx Ca IIA	RAH + PLND (1992)	Acquired	Bilateral	WLE (2009)	Improved	Improved	Improved	Improved	Absent	Recurred (2014)/observation
2	62/60	Edema, pain	Cx Ca IA	RAH + PLND + RT (1991)	Acquired	Bilateral	WLE (2012)	Improved	Absent	Improved	Absent	Absent	No recurrence
3	54/51	Pruritus, edema, discharge	Cx Ca IB	RAH + PLND + RT (2000)	Acquired	Right	LE (2012)	Absent	Stationary	Improved	Improved	Absent	Recurred in left vulva (2012)/WLE
4	36/35	Pain, edema, infection	Cx Ca IIA	RAH + PLND + CCRT (2006), RT (2009)	Acquired	Bilateral	WLE (2013)	Improved	Absent	Improved	Absent	Improved	No recurrence
5	63/60	Pain, discharge	Cx Ca IB	RAH + PLND (1999)	Acquired	Bilateral	WLE (2013)	Improved	Absent	Absent	Improved	Absent	No recurrence
6	77/73	Pain, pruritus	Cx Ca IB	RAH + PLND + RT (1994)	Acquired	Bilateral	WLE (2013)	Improved	Stationary	Absent	Absent	Absent	No recurrence
7	61/56	Pain, edema, discharge	Cx Ca IIA	RAH + PLND + CCRT (1998)	Acquired	Bilateral	WLE (2014)	Improved	Absent	Improved	Improved	Absent	No recurrence
8	60/52	Pain, edema	Cx Ca IIA	RAH + PLND + RT (1983)	Acquired	Bilateral	WLE (2014)	Improved	Improved	Absent	Absent	Absent	No recurrence

*Cx Ca* cervical cancer, *LC* lymphangioma circumscriptum, *RAH* radical abdominal hysterectomy, *PLND* pelvic lymph node dissection, *WLE* wide local excision, *RT* radiation therapy, *CCRT* concurrent chemoradiation therapy, *LE* local excision

**Fig. 1:**
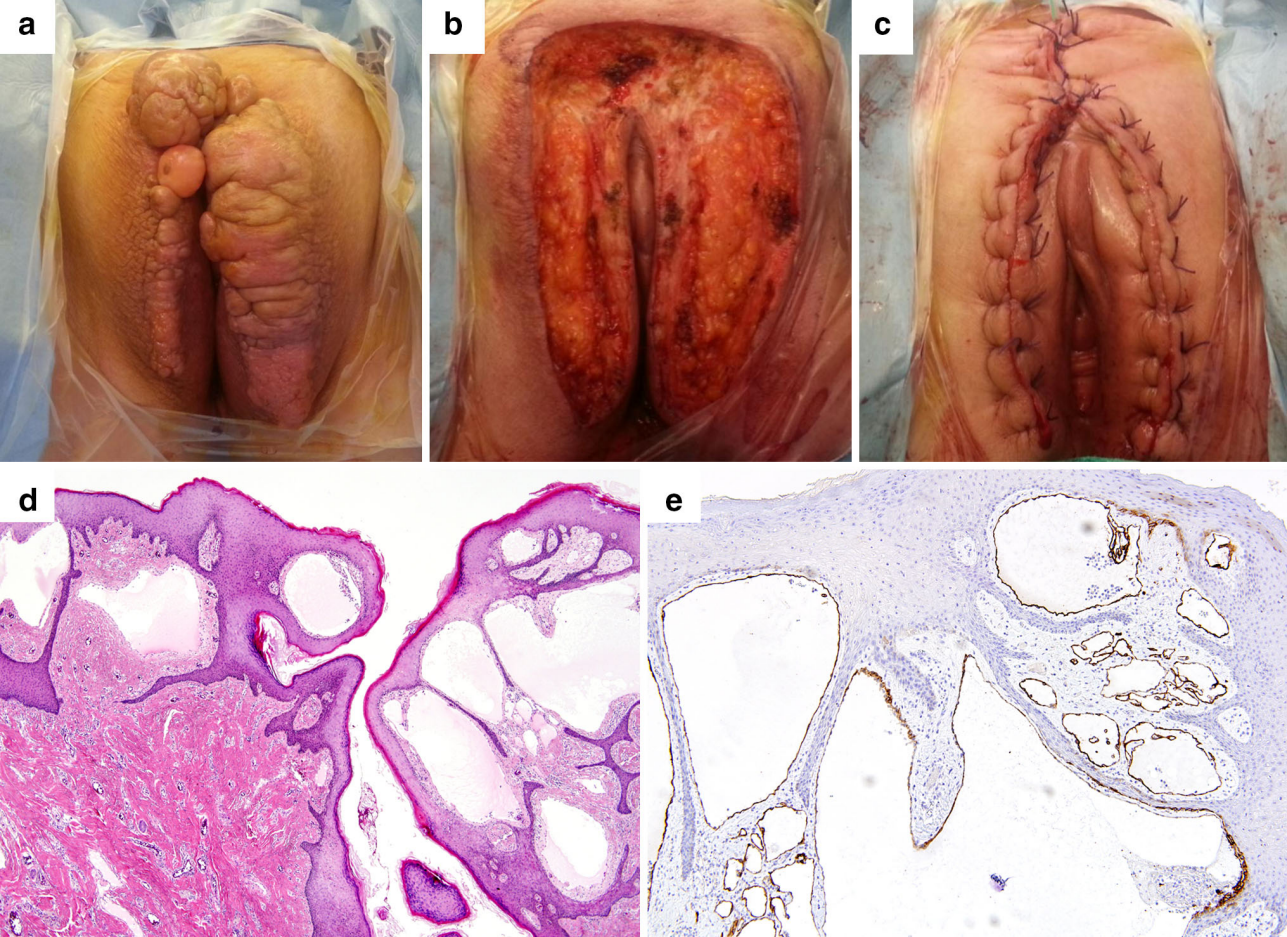
Intraoperative and postoperative photographs (**a**–**c**) and microscopic findings (**d**, **e**) of vulvar LC (patient 8). **a** Gross finding. **b** Right after the surgical excision. **c** Postoperative finding. **d** Histopathologic examination revealed hyperkeratotic, hyperplastic squamous epithelium in the epidermis and multiple, variable-sized spaces lined by flat endothelial cells in the superficial dermis. The dilated dermal lymphatic channels contain fibrinous material and few inflammatory cells. **e** Immunohistochemically, the lymphatic endothelial cells were positive for D2-40 [1]

Intraoperative gross photographs (Fig. 1a, b), a postoperative photograph (Fig. 1c) and histopathologic findings of patient 8 are shown. Gross examination revealed hyperpigmented, rugose and studded epidermis with multiple conglomerated papules measuring up to 0.1–0.5 cm. The cut sections showed nodularity and papillary projections with thin-walled cystic cavities in the superficial dermis. Microscopic examination revealed multiple dilated dermal vascular channels containing eosinophilic proteinaceous material (Fig. 1d). The lymphatic channels were lined by a single layer of bland endothelial cells and highlighted by D2-40 immunostaining (Fig. 1e). There was a mild inflammatory infiltrate in the upper dermis. The overlying epidermis was partly hyperkeratotic. There was no evidence of malignancy.

Most chief complaints showed considerable improvements on assessment at the outpatient clinic after the primary surgery. No patient showed an aggravation of the symptoms. Two patients developed local recurrences: patient 1 developed recurrence 47 months after the primary surgery. Since the lesion was very small and localized, we decided to manage it conservatively, but monitor it very closely. Patient 3 developed recurrence on the opposite side 13 months after local excision. We performed a second wide local excision. The remaining six patients remained free of recurrence.

## Discussion

We performed a thorough search for previous literature on the treatment of vulvar LC using the US National Library of Medicine’s PubMed database and the Royal College of Obstetricians and Gynecologists database using the term “lymphangioma circumscriptum” and the word “vulva”. Among 44 reported cases (in any language) of vulvar LC by December 2014, only 24 cases acquired LC. Out of the 24 previous cases of LC, 21 cases were associated with treatment of cervical cancer (87.5 %). In the remaining three cases, patients had Crohn’s disease, recurrent cellulitis, and leg edema. To the best of our knowledge, this report exhibits the largest number of case series from a single institution so far.

The etiology of acquired LC remains to be clarified. The suggested etiology is the architectural disruption of previously normal lymphatic channels, leading to LC by the sequestration and further dilation of previously normal lymphatics [7, 8]. Causative factors of vulvar-acquired LC are radical hysterectomy, pelvic lymphadenectomy, and/or radiation therapy for cervical cancer, infectious disease (filariasis, genital tuberculosis, erysipelas, sexually transmitted disease, and lymphogranuloma venereum), Crohn disease, primary dysplastic angiopathy, bilateral varicose veins in the lower extremities, dermopathy due to penicillamine or corticosteroids, surgical trauma, keloids, scleroderma, and rhabdomyosarcoma [9]. If the patient has no causative factor, the diagnosis of primary vulvar LC is made. However, this is an extremely rare condition [10]. According to our experiences, the radicality of surgical treatment seems to have more impact on the development of vulvar LC than the tumor stage. Indeed, there may be a tendency that the surgical radicality increases as the tumor stage gets high. Nevertheless, considering the mechanism by which surgical procedure damages the lymphatic vascular channels, we think that vulvar LC occurs more frequently in patients who received radical surgery than in those who did not receive radical surgery. Regarding the type of therapy chosen for cervical cancer, our patients received different options of treatment from each other, including surgery only, surgery, adjuvant chemotherapy, and/or radiation therapy, since the patients were treated by different clinicians and each of them had different treatment policies. Even though some subgroups of patients exhibited the same clinical stage (IIA in 4 patients and IB in 3), correlating the type of therapy with the presence of vulvar LC seems to be inadequate in this study.

Differential diagnoses of vulvar LC include molluscum contagiosum, herpes zoster, genital warts, lupus verrucosus, leiomyoma, cellular angiofibroma, angiomyofibroblastoma, and aggressive angiomyxoma [11, 12]. As described above, the clinical manifestation of various infectious disease and tumorous conditions are similar to that of vulvar LC. Therefore, the histopathologic confirmation of diagnosis through biopsy is crucial to avoid misdiagnosis and mistreatment. In addition, the correct diagnosis is essential to determine the optimal therapeutic strategy, which may satisfy both patients and clinicians.

There is no consensus about the standard treatment for vulvar LC. Treatment modalities reported in the literature include surgical excision, abrasive methods (CO_2_ laser, liquid nitrogen, electrocoagulation or sclerosing therapy), and observation. There are several therapeutic options for surgical excision, such as labiaectomy, vulvectomy (simple, partial, or radical), mass excision, and wide local excision. In general, it is conducted to reduce labium majora and minora, as well as to excise vulvar verrucous and edematous lesions as much as possible. A previous study reported that with a single surgery for vulvar LC, at least 10 years of disease-free, long-term cure is possible [13]. Several authors have proposed that the most preferred treatment of choice for both primary and acquired LC is surgical excision [10]. The age of presentation is relatively low, and considerable cosmetic problems affect patients physically and psychologically. In this study, six of eight patients developed no recurrence. One of two patients who developed local recurrence received second complete surgical excision. Consistent with previous data, we also propose that surgical excision is a treatment of choice for vulvar acquired LC. Indeed, it is not proven to be superior compared to other treatment modalities [8]. Ghaemmaghami et al. [12] reported the recurrence rate after surgery was 23.1 % during follow-up periods, ranging from 6 to 81 months. Vlastos et al. [14] stated that the postoperative recurrence rate might be twice as high in LC without surgical treatment. To prevent developing recurrence, excision of the lesion should be through the full thickness of the skin and subcutaneous tissue down to the deep fascia [11]. By doing this, the deep-feeding lymphatic cisterns of subcutaneous layer, which are considered to be the main cause of the recurrence, can be excised. We assume that the extent of the primary lesion has significant impact on recurrence and surgical outcome. Treatments should be individualized according to the extent, type, and severity of disease and patient’s preference.

Non-surgical treatment including cryotherapy, sclerotherapy, and laser therapy have been attempted to prevent surgery-related complications. Actually, in clinical practice, CO_2_ laser, electrocoagulation, or sclerosing therapy has been performed for local therapy. Though in some cases, those treatment options have been reported to be effective in controlling the symptoms, a thorough literature search revealed negative results. There were no available data regarding the effectiveness of local therapy for vulvar LC using a large-scale patient cohort. As a result, currently, no local therapy has been proved to reliably improve symptoms such as pain and/or pruritus. Cryotherapy seems to be rather ineffective, with low remission and high recurrence rates. A previous study showed that sclerotherapy is very effective in a short term [15], but sclerotherapy agents have a potential risk of severe systemic, local, and cosmetic side effects. In contrast, regarding CO_2_ laser therapy, the effectiveness of pulsed dye lasers was reported in 2005 [16]. Favorable outcomes using a 900-nm diode laser and CO_2_ laser were also reported in 2006 [17]. Similarly, successful treatment of congenital vulvar LC with CO_2_ and long-pulsed Nd:YAG lasers has been recently reported [18]. More clinical data on laser therapy are necessary. We believe that it will be favorable to consider surgical treatments when fulfilling the following criteria: (1) the large size of mass and deep lesions of vulvar LC; (2) distressing symptoms such as pain, pruritus, edema, discharge, and secondary infection; (3) treatment failure after non-surgical treatment.

A limitation of this study was the relatively small sample size and the lack of a comparison group, i.e., patients who did not receive non-surgical treatment. It would be interesting to observe patients who received medical treatment and compare the results. We considered that this study is just a preliminary one to analyze the clinical outcomes of surgical treatment for vulvar LC. We are now collecting the data on non-surgical treatment for vulvar LC. We are planning to report the experience of non-surgical treatment for vulvar LC and compare the results between patients who received radical surgery and those who did not.

In conclusion, we assessed the clinical outcomes of primary surgical treatment for patients with vulvar LC. Because there are a number of diseases that exhibit similar clinical manifestation to that of vulvar LC, it is not easy for gynecologists to have an initial clinical diagnosis. Even if it is diagnosed correctly, relapse often occurs. Relevant symptoms are not only distressing, but also affects patients’ quality of life. The treatment modalities may differ depending on patients’ age, the extent of the lesion, and the preference of each patient and clinician. Nevertheless, we propose that surgical treatment could provide a more long-lasting answer compared to other treatment modalities, since it is beneficial in terms of clinical outcomes. In the future, a long-term follow-up investigation is required to assess the prognosis and to compare the efficacy and side effects of each modality.
